# Secular Increasing Trends in Female Thyroid Cancer Incidence in Taiwan

**DOI:** 10.3390/life14070809

**Published:** 2024-06-26

**Authors:** Jiun-Yan Wu, Yuh-Kae Shyu, Yu-Kwang Lee, Yu-Chiao Wang, Chun-Ju Chiang, San-Lin You, Li-Jen Liao, Wan-Lun Hsu, Yong-Chen Chen

**Affiliations:** 1 School of Medicine, College of Medicine, Fu-Jen Catholic University, New Taipei City 242, Taiwan; alvinwu900225@gmail.com (J.-Y.W.); 087780@mail.fju.edu.tw (S.-L.Y.); 2 Department of Nursing, College of Medicine, Fu-Jen Catholic University, New Taipei City 242, Taiwan; 094528@mail.fju.edu.tw; 3 Division of General Surgery, Department of Surgery, National Taiwan University Hospital, Taipei 100, Taiwan; viviankwang125@gmail.com; 4 Master Program of Big Data in Medical Healthcare Industry, College of Medicine, Fu Jen Catholic University, New Taipei City 242, Taiwan; 406088139@mail.fju.edu.tw; 5 Graduate Institute of Epidemiology and Preventive Medicine, College of Public Health, National Taiwan University, Taipei 100, Taiwan; ruru.chiang@cph.ntu.edu.tw; 6 Data Science Center, College of Medicine, Fu Jen Catholic University, New Taipei City 242, Taiwan; 7 Otolaryngology Head and Neck Surgery, Far Eastern Memorial Hospital, Taipei 100, Taiwan; deniro@mail2000.com.tw; 8 Head and Neck Cancer Surveillance and Research Group, Far Eastern Memorial Hospital, New Taipei City 242, Taiwan; 9 Department of Electrical Engineering, Yuan Ze University, Taoyuan 320, Taiwan

**Keywords:** thyroid cancer, incidence, fertility, overdiagnosis, obesity

## Abstract

Background: Thyroid cancer incidence has increased globally in recent decades, especially in females, although its trends in Taiwan have not been studied extensively. This study aimed to investigate changes in female incidence and possible causes of thyroid cancer in Taiwan. Methods: Using the Taiwan Cancer Registry (TCR) Database, age-standardized incidence rates, age-specific incidence rates and birth cohorts were calculated. Correlation between female thyroid cancer incidence and cohort fertility rates were examined. Results: Thyroid cancer incidence increased in Taiwanese female, with age-adjusted rates per 100,000 people increasing from 7.37 during 1995–1999 to 20.53 during 2015–2019; the annual percentage change (APC) was 5.9% (95% CI, 5.3–6.5). Age-specific incidence rates increased with age, with peak rates occurring at younger ages. The APCs in the 50–54 age group were the highest (6.8%, 95% CI, 6.1–7.5). Incidence rates also increased with later birth cohorts. We observed a significant negative correlation between thyroid cancer incidence and fertility rates in the same birth cohort. Conclusions: We hypothesize that overdiagnosis may be a main reason for the rapidly increasing thyroid cancer incidence in Taiwanese females. Notably, we observed a strong negative correlation between fertility and thyroid cancer incidence. However, our study is limited by the absence of individual-level cancer data in the TCR database. These associations with fertility will be an important subject for future thyroid cancer research.

## 1. Introduction

Thyroid cancer is a malignant tumor that arises from either the thyrocytes or the parafollicular cells of the thyroid gland. It usually appears as one or more firm to hard nodules in the thyroid, and approximately 5% of detected thyroid nodules are malignant [1]. Thyroid cancer incidence has risen globally over the past few decades, increasing from 95,030 incident cases in 1990 (95% confidence interval [CI], 90,070–100,720), to 255,490 cases in 2017 (95% CI, 245,710–272,470) [2], and the number was estimated to be 586,202 in 2020 [3]. In the USA, an estimated 44,020 new cases are expected in 2024. (https://cancerstatisticscenter.cancer.org/types/thyroid, accessed on 20 June 2024). Incidence rates differ by sex and show an approximate 3:1 ratio comparing rates between females and males [4,5].

Although thyroid cancer incidence in various countries is generally on the rise and the increase is higher for females than for males, the incidence and trend patterns vary from country to country and can be roughly divided into several groups. Thyroid cancer incidence is relatively high and continues to rise in North America. In the United States, the incidence rate per 100,000 people increased from 5.3 in females and 2.2 in males in 1982 to 17.2 in females and 5.8 in males in 2016. In China and South Korea, incidence rates increased rapidly after the 21st century. In South Korea, the incidence rate per 100,000 people increased from 10.3 in females and 2.1 in males in 2000 to 110.7 in females and 25.3 in males in 2012. In northern European countries, the incidence rate is relatively low and has risen less dramatically. In Sweden, the incidence rate per 100,000 people increased from 4.34 in females and 1.8 in males in 1980 to 6.4 in females and 2.0 in males in 2016. The incidence rate data mentioned above are sourced from the Global Cancer Observatory website (https://pse.is/5kgwe6, accessed on 20 June 2024). Thyroid cancer incidence rates have also continued to rise in Taiwan, and the age-standardized rate in females increased from 4.39 in 1993–1996 to 17.29 in 2013–2016 per 100,000 individuals [6].

Thyroid cancer is a disease with multiple risk factors [7]. Incidence is three times higher in females compared to males, suggesting female sex hormones may play a role [8]. These hormones have been found to be related with the pathogenesis of breast, prostate, endometrium, ovarian, and testicular cancers [9], and estrogen exposure is a verified risk factor for some hormone-related cancers, such as breast [10] and endometrial cancer [11]. Estrogen has been found to impact the proliferation and growth of malignant thyroid cells [12,13], which may be related with its receptors [14,15] or microenvironment change [16]. Females with a history of breast cancer are approximately three times more likely to develop thyroid cancer compared to other females (relative risk (RR) = 2.7, 95% CI, 0.78–7.9) [17]. During pregnancy, the expression of estrogen and its receptors in the mother changes, and pregnancy has been found to have a protective effect against breast cancer. [18]. In comparison, lower parity in pregnancy has been found to have a protective effect against breast cancer [19]. It is worth investigating whether similar effects may occur for thyroid cancer. Furthermore, late age at menopause also appears to be associated with an increased risk of thyroid cancer [19].

Previous research has found that exposure to phthalates is a risk factor for thyroid nodule and thyroid cancer [20]. In addition, the risk of thyroid cancer increases with BMI [21], and conditions such as type 2 diabetes [22] and iodine deficiency [23]. According to the Nutrition and Health Survey in Taiwan (NAHSIT), the proportion of Taiwanese people who are overweight or obese, as well as the incidence of type 2 diabetes, has increased over time. The proportion of Taiwanese people with iodine deficiency has also been changing [24]. Exposure to ionizing radiation, especially during childhood, is another risk factor [25]. Radiation-induced DNA damage and subsequent mutations can disrupt the normal regulatory mechanisms, leading to uncontrolled cell growth and the development of malignant tumors. Additionally, advancing age is associated with an increased risk [2], indicating a cumulative effect of genetic and environmental factors over time. Understanding of the molecular basis of thyroid cancer has revealed specific genetic alterations associated with different subtypes. For instance, papillary carcinoma often harbors BRAF and RET/PTC mutations, while follicular carcinoma may involve RAS mutations. Medullary carcinoma is associated with mutations in the RET proto-oncogene [26].

Factors influencing the prognosis of thyroid cancer include not only staging but also the type of cancer cells. Thyroid cancer can mainly be categorized into follicular-derived thyroid cancers and neuroendocrine C-cell-derived thyroid cancers (medullary carcinoma) [27]. Follicular-derived thyroid cancers can be categorized into papillary carcinoma, follicular carcinoma, and anaplastic carcinoma [28]. In Taiwan, the overall 2-year survival rate for thyroid cancer in 2012 was 96.1%. However, there are significant differences in mortality rates among the subtypes: the 2-year overall survival rates in 2012 were 98.0%, 95.1%, 89.7%, and 6.9% for these subtypes, respectively [29].

Thyroid cancer incidence in Taiwan has risen rapidly in 1995 to 2019 [30], especially in females. However, there is currently limited exploration of age effect, period effect, and cohort effect in the existing studies. As exposure to its risk factors has also changed significantly, the impact of these changes on thyroid cancer incidence is still unclear. Thus, this study aims to describe the time-dependent characteristics of female thyroid cancer incidence in Taiwan.

## 2. Materials and Methods

We used the Taiwan Cancer Registry (TCR) data to calculate thyroid cancer incidence among Taiwanese females from 1995 to 2019, using the following codes: ICD-O-FT: T-193 and ICD-O-3: C73. We identified 43,751 cases of thyroid cancer during the study period. The TCR is a highly complete and accurate database with a coverage rate of 98.4% [31]. According to the TCR Report (https://pse.is/5kgsxw, accessed on 20 June 2024), the proportion of patients with thyroid cancer confirmed by cytology or histopathology has been consistently high, rising from 93.60% in 1995 to 98.27% in 2000 and then to 99.61% in 2019.

Age-specific incidence rates were grouped every five years, from age 30 to 89, for a total of 12 age groups (30–34, 35–39, 40–44, …, 80–84, 85–89). Diagnosis periods were also grouped every five years, from 1995 to 2019, for a total of 5 periods (1995–1999, 2000–2004, 2005–2009, 2010–2014, 2015–2019). Birth cohorts were grouped every five years from 1925 to 1974 for a total of 10 groups (1925–1929, 1930–1934, 1935–1939, …, 1965–1969, 1970–1974). Age-specific incidence rates by period and birth cohorts were provided. In order to facilitate a direct comparison of rates across different countries, we employed the World Health Organization (WHO) 2000 World Standard Population to calculate the age-standardized incidence rates. To assess temporal trends and changes in data over time, we calculated the annual percentage change (APC) in thyroid cancer incidence rates, including their respective 95% confidence intervals. This analysis utilized the National Cancer Institute’s (NCI) Joinpoint program (version 4.9.1.0).

As Taiwanese law requires citizens to declare and provide birth data, we collected fertility rate data from 1950 to 1999 from the National Development Council and Department of Household Registration websites to examine the relationship between fertility and thyroid cancer incidence. Considering the incubation period between fertility and thyroid cancer, we performed a Spearman rank correlation analysis between thyroid cancer incidence rates among 40–59-year-old females and fertility rates of 20–29-year-old females in the same birth cohort (1945–1949, 1950–1954, 1955–1959, 1960–1964, and 1965–1969). The closer the r value is to 1 or −1, the greater the positive or negative correlation.

The data for correlating thyroid cancer incidence rates and fertility rates across different countries were gathered form cancer incidence in five continents (CI5) and the world bank (https://pse.is/5kgtcz, accessed on 20 June 2024). For the purpose of conducting international comparisons, we chose to analyze using age-standardized incidence rates and total fertility rates. We utilized data from volumes 4, 9, and 12 for 5-year periods of age-standardized incidence rates: 1973–1977, 1993–1997, and 2013–2017, respectively. The selected countries include Europe (United Kingdom, Sweden, and Finland), North America (United States and SEER), Oceania (Australia), and Asia (Hong Kong, Japan, and Singapore). Based on the causal continuity of the disease, fertility rates were selected for the years 1960, 1980, and 2010. This research protocol was approved by the Institutional Review Board of Fu-Jen Catholic University (No. C110216). This study was performed in accordance with the Declaration of Helsinki. Patients or the public were not involved in this study.

## 3. Results

**Figure 1:**
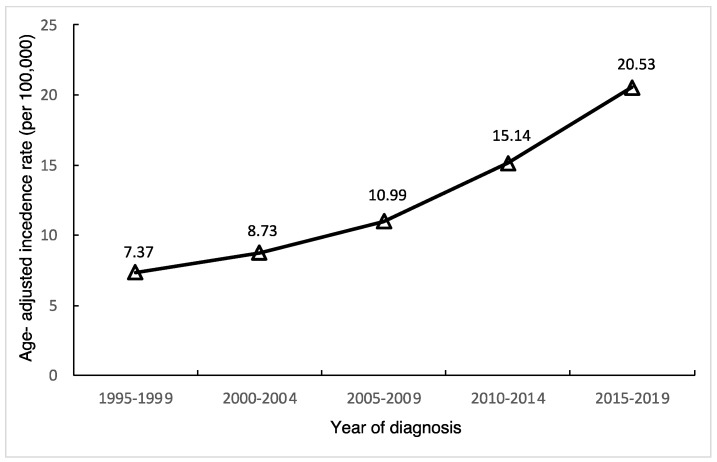
Age–standardized incidence rate of thyroid cancer in Taiwanese females, 1995–2019.

After calculating age-standardized incidence rates of thyroid cancer in Taiwan, we found the age-standardized incidence in females continually rising (Figure 1), from 7.37 per 100,000 during 1995–1999 to 20.53 per 100,000 during 2015–2019, with an APC of 5.9% (95% CI, 5.3–6.5) as shown in Table 1. The relative percent change from 1995–2019 was 272.9%.

**Figure 2:**
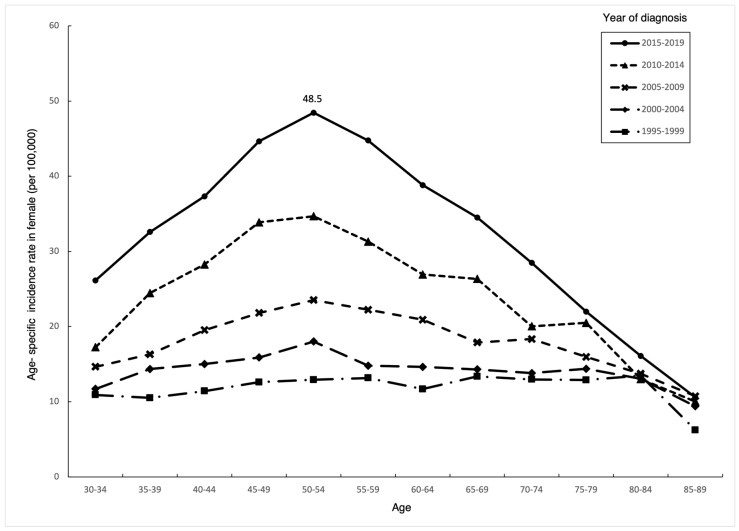
Age–specific incidence rates of thyroid cancer among Taiwanese females aged 30–89 by year of diagnosis, 1995–2019.

Thyroid cancer incidence increased over time in all age groups of females (Figure 2), and the magnitude increased over time. The age-specific incidence among females generally increased with age, reaching a peak of 48.5/100,000 during 2015–2019 in the 50–54 age group, then rapidly decreased with age, thereafter, resulting in an overall trend resembling an inverted “V” shape. In comparison, in the early cohorts (1995–1999), there is no significant peak of thyroid cancer incidence. Table 1 shows that the highest APC occurred in the 50–54 age group (6.8%, 95% CI, 6.1–7.5), while the APC in individuals over 75 years old was less than 3%.

**Figure 3:**
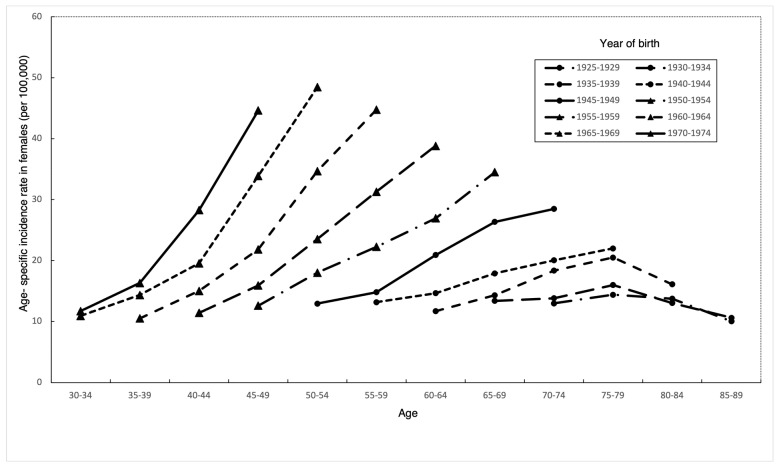
Age–specific incidence rates of thyroid cancer among Taiwanese females aged 30–89 by year of birth, 1995–2019.

In every birth cohort, thyroid cancer incidence increased with age (Figure 3). Incidence rates in early birth cohorts increased with age, reached a peak at ages 70–74, and declined thereafter. The highest incidence occurred at increasingly younger ages in later birth cohorts. In addition, we observed a trend of accelerating age-specific incidence rates in different birth cohorts. That is, individuals born in later birth cohorts were reaching the same incidence rate at earlier points in life. In addition, there were fewer changes in incidence among cohorts prior to 1944 compared to cohorts after 1945–1949.

**Figure 4:**
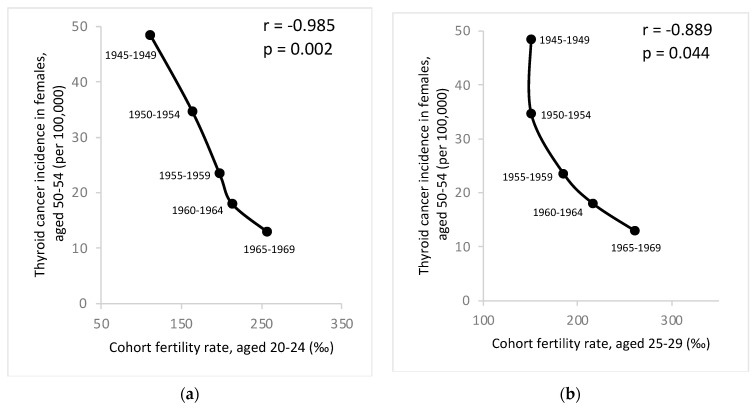
(**a**) Correlation between age–specific thyroid cancer incidence rates in females aged 50–54 and cohort fertility rates in females aged 20–24 in Taiwan. (**b**) Correlation between age–specific thyroid cancer incidence rates in females aged 50–54 and cohort fertility rates in females aged 25–29 in Taiwan.

Figure 4 shows a significant negative correlation between thyroid cancer incidence in the 50–54 age group and fertility rates in the 20–29 age group among females from the same birth cohort. The age-specific fertility rates in Taiwanese females from 1950–1999 are detailed in Supplementary Table S1. The R values are −0.985 (*p* = 0.002) in the 20–24 age group and −0.889 (*p* = 0.044) in the 25–29 age group, both of which are statistically significant. This suggests that fertility rates may play a role in the dramatic changes in thyroid cancer incidence among Taiwanese females in recent decades. Similar graphs showing strong correlation between thyroid cancer incidence in females of other age groups (40–59 years) and fertility rates in females aged 20–29 years from the same cohort can be found in Supplementary Figure S1.

The relationship between thyroid cancer incidence rates and fertility rates across different countries is shown illustrated in Supplementary Figure S2. Within each region, there is a consistent pattern of thyroid cancer incidence generally increasing over time, while fertility rates concurrently decrease, indicating a negative correlation between the two variables. Regional trends, however, exhibit some variations: in Asia, there is a rapid decline in fertility rates coupled with a notable increase in thyroid cancer incidence. In the Americas, thyroid cancer incidence has also experienced a rapid rise, although the decline in fertility rate is not as pronounced. Meanwhile, in Europe, changes in both thyroid cancer and fertility rates appear to be relatively gradual.

## 4. Discussion

Thyroid cancer incidence in Taiwanese female was rising rapidly during 1995–2019, and the largest increase occurred in the 50–54 age group. After investigating changes in Taiwan’s fertility rates, we found a strong negative correlation between fertility rates and female thyroid cancer incidence. In addition, thyroid cancer incidence increased with later diagnosis years and birth cohorts. We consider that some possible causes for the changing thyroid cancer incidence can be divided into two categories: increases due to overdiagnosis or actual increases in thyroid cancer incidence. Actual increases may be associated with changes in many risk factors, including fertility, plastic exposure, diabetes, obesity, iodine, radiation, and occupation. These may relate with hormonal and reproductive factors.

### 4.1. Overdiagnosis

In the early cohorts of age-specific incidence among females, there is no significant peak of thyroid cancer incidence. However, as time progresses, the overall trend of an inverted “V” shape becomes increasingly evident, with the peak occurring in the 50–54 age group. We hypothesize that, due to increased screening, female patients who were diagnosed at a later age in earlier cohorts are now being diagnosed at an earlier age. Additionally, commercial health check-ups in Taiwan typically start around this age, which may be related to increased public awareness. Another possible factor is changes in fertility rates, as this group of women represents the population that experienced a significant decline in fertility following World War II.

As medical technology improves, health examinations and cancer screenings have become increasingly sophisticated. However, thyroid cancer overdiagnosis has been a global health problem [32]. In many countries, including the United States, South Korea, and European countries, overdiagnosis is considered a main reason for the increase in thyroid cancer incidence. Research estimates that during 2003–2007, overdiagnosis accounted for 90% of thyroid cancer cases in South Korea and 70–80% of cases in the United States [33].

Since thyroid cancer typically manifests as firm to hard thyroid nodule (or nodules), initial evaluation of suspected patients often includes thyroid ultrasound. In the United States, the number of Medicare claims with thyroid ultrasound as an initial imaging test increased 20.9% per year from 2002 to 2013 [34]. In South Korea, the proportion of new cases diagnosed by ultrasound screening increased rapidly from 13.0% in 1999 to 56.7% in 2008. The increased use of ultrasound correlates with the rapid increase in thyroid cancer incidence in South Korea [35], and we may be seeing a similar situation in Taiwan. Taiwan’s national health insurance (NHI) program was established in 1995. Although it allows doctors to have access to better tools, such as ultrasound, for more extensive diagnostic capabilities, it also leads to early diagnosis and possible overdiagnosis. According to one study that randomly sampled one million people from the NHI report (2004–2010), there was a 440.1% increase in the number of individuals who received ultrasound-guided fine-needle aspiration biopsy of the thyroid in Taiwan, most of which were ordered by internal medicine physicians (76.4%). During this same time period, the age-standardized incidence rate of thyroid cancer increased by 94.8% [36]. In addition, changes in diagnostic criteria may also have an impact. For instance, between 2009 and 2017, the American Thyroid Association (ATA) strongly advised against biopsies for extremely small thyroid nodules without suspicious characteristics and discouraged the screening of thyroid cancer in asymptomatic individuals [37]. Tumor size and clinical staging data at the time of diagnosis also showed a trend toward earlier diagnosis; thyroid cancer patients diagnosed during 1993–1998 were diagnosed earlier than those diagnosed during 1979–1992 [38]. Furthermore, the change in the proportion of early thyroid cancer diagnoses after the NHI program was established in 1995 is also worth exploring.

Among the four histological subtypes of thyroid cancer, the overall rates, ranked from highest to lowest, are as follows: papillary carcinoma, follicular carcinoma, medullary carcinoma, and anaplastic carcinoma. Based on a study conducted by Ahn et al., thyroid cancer screening shows a strong association with the rise in the papillary carcinoma subtype [39]. According to the TCR annual report (pse.is/5kgsxw), the majority of the increase in thyroid cancer incidence in Taiwan was due to papillary carcinoma. Out of all thyroid cancers, the proportion of papillary carcinoma for females increased from 76.78% in 1995 to 92.60% in 2019. This change may be due to the increase in the proportion of Taiwanese patients undergoing opportunistic thyroid cancer screening.

### 4.2. Fertility

We observed that thyroid cancer incidence increased significantly in females aged 50 to 54 and a significant negative correlation between thyroid cancer incidence in the 50–54 age group and fertility rates among females from the same birth cohort in the 20–29 age group. Our hypothesis suggests a potential association with fertility rates.

Estrogen promotes cancer cell progression by modulating signaling pathways, such as the MAPK and PI3K/Akt pathways, leading to increased cell proliferation and survival [40]. Estrogen’s impact on thyroid cancer may be related to the regulation of estrogen receptors. In thyroid cancer cells, the concentrations of estrogen receptor alpha (Erα) and beta (Erβ) were found to be increasing [41], and their genes were found to be overexpressed. In addition, ERα activation promoted cell proliferation, angiogenesis, and migration via pathways such as AKT/mTOR, MEK1/2, and MAPK [42].

In previous studies, we found similarities in the impact of fertility on thyroid and breast cancers. Studies found increased odds of thyroid cancer in females with a late age at first birth [43]. Compared with females whose first birth took place at <20 years old, females who experienced their first childbirth at ≥35 years old had a 2.7 (1.1–6.8)-fold risk of developing thyroid cancer. In comparison, late age at first birth has been found associated with increasing risk of developing breast cancer in many studies [43,44], and a negative correlation has been found between the number of deliveries and breast cancer, with an OR of 0.81 (95% CI, 0.7–0.9) [45,46]. Similarly, our results also showed increased thyroid cancer incidence after the age of 40 in a low-fertility female cohort. These results suggest that the recent decline in Taiwan’s fertility rates may be correlated with the substantial increase thyroid cancer incidence among Taiwanese females.

Moreover, the reduction in fertility rates is intertwined with global social and economic dynamics. Initially, economic growth aligns with higher fertility rates, but as economic expansion accelerates, fertility rates decline [47]. On the other hand, thyroid cancer incidence shows a strong correlation with national development and health expenditures across countries. The human development index (HDI) was correlated with thyroid cancer incidence (β  =  0.523, 95% CI  =  0.275–0.771) [48]. However, we do not have sufficient evidence to determine if this conclusion is not independent of other factors such as overdiagnosis.

Therefore, fertility may not be an independent factor influencing thyroid cancer, possibly excluding it from considerations of overdiagnosis. In addition, pregnancy is generally not a risk factor for thyroid cancer recurrence. There was no difference in recurrence rates between women who became pregnant after their initial diagnosis and those who did not [49]. Therefore, it is unlikely that women over 35 years old will avoid pregnancy due to an increased risk of developing thyroid cancer. On the other hand, there has been evidence that women with infertility are at an increased risk of developing thyroid cancer [50].

In addition, we further speculate that the same phenomenon may also be occurring in other countries with rapid changes in fertility rates, not only in Asia and America, but also Europe, and this will be an important health issue in the future. However, to our knowledge, no relevant molecular or biological research results have been found, and we are unable to explain or verify its biological mechanism.

### 4.3. Plastic Exposure (Phthalates)

Phthalates are risk factors for thyroid cancer because they can mimic thyroid hormones and disrupt thyroid function [51]. Di-(2-ethylhexyl) phthalate (DEHP) exposure in mice can cause thyroid cell proliferation and DNA damage and activates the thyroid stimulating hormone receptor pathway [52]. Taiwan has a thriving plastic industry. In 2000, the concentrations in the surface water of two major phthalates ranged from ND to 18.5 μg/L for DEHP and from 1.0–13.5 μg/L for di-*n*-butyl phthalate (DBP) [53]. In comparison, Europe has a relatively low thyroid cancer incidence, and the total concentration of eight phthalates (including DHEP, DBP) in 2014 was 1.2 μg/L in the Rhone River, a major river crossing France and Switzerland [54]. The concentration of phthalates in Taiwanese rivers is much higher than in Europe. Therefore, the higher concentration of phthalates Taiwanese environment may be one of factors contributing to the rapid increase in thyroid cancer. Additionally, research has revealed that in the year 2012–2013, 11% of children younger than 12 years old and 1% of adults in Taiwan were highly exposed to DEHP (>100 μg/kg_bw/day). Moreover, 15% of children had high average daily intakes of DEHP (ranging from 50 to 100 μg/kg_bw/day) [55]. However, there is currently a lack of research on the concentration of phthalates in the blood, and more evidence is needed to speculate on their effects on the population in Taiwan.

### 4.4. Diabetes Mellitus

Individuals with diabetes have an increased risk of thyroid cancer, with an HR of 1.38 (95% CI, 1.13–1.67) for females [22]. Thus, the rapid increase in diabetic patients in Taiwan may be one explanation for the rising thyroid cancer incidence in females. From 2005–2014, the incidence of diabetes increased by 19%, and the increase was most obvious in patients aged 20–39 years [24]. Additionally, the rise in thyroid cancer incidence was more pronounced among younger age groups, suggesting a potential correlation between the two. Moreover, a study revealed that the use of metformin in individuals with type 2 diabetes might lower the risk of thyroid cancer [56]. Although the association between diabetes and thyroid cancer is still unclear, this observed trend underscores the need for further research.

### 4.5. Obesity

Obesity is closely related to thyroid and sex hormones. Serum thyroid-stimulating hormone and T3 were found to be positively associated with BMI [57]. Body fat distribution and adipocyte differentiation were influenced greatly by sex hormones [58], and both sex hormones and sex hormone-binding globulin were found to have a strong correlation with obesity [59]. Obesity was found to be a risk factor for thyroid cancer, and a meta-analysis of 282,137 people found that obesity has a greater impact on thyroid cancer in females [60] (per 5 kg/m^2^: HR = 1.14 [95% CI, 1.06–1.23] in females). According to the Nutrition and Health Survey in Taiwan (NAHSIT), the rate of obesity (BMI > 24) in Taiwanese people has increased from 33.0% in 1993–1996 to 42.8% in 2017–2020 among females.

### 4.6. Other Factors: Iodine, Radiation, and Occupational Risk Factors

Iodine deficiency is a risk factor for benign thyroid disorders, such as goiter, nodules, and thyroid cancer [23]. According to NAHSIT, the prevalence rate of iodine deficiency (urine iodine < 100 ug/L) during 2014–2016 was 25.8–33.8% in females. Notably, in 2017, the government raised the standard for iodized salt addition from 12–20 to 20–33 mg/kg, and the prevalence rate of iodine deficiency subsequently decreased to 18.6–25.0% in females during 2017–2019.

Radiation is also a risk factor for thyroid cancer, and the risk increases with higher radiation doses (>100 mSv/year) or younger age at exposure, and the risk also significantly decreases as the age at exposure increases, with minimal risk observed after the age of 20 years [25]. The radiation exposure that the general population receives primarily falls into two categories: medical radiation and background radiation. In Taiwan, the average dose of medical radiation per person rose from 0.81 mSv/yr in 1992–1994 to 3.44 mSv/yr in 2021. As the background radiation dose in Taiwan is about 2 mSv/yr [61], people who exceed the safe dose should be in the minority.

Thyroid cancer has a higher prevalence rate among health practitioners, nurses, caregivers, cleaners, pest control workers, chefs, and the retail industry [62]. However, these groups only account for a small proportion of Taiwan’s population and should not be a major reason for the massive increase in thyroid cancer incidence in Taiwanese females.

### 4.7. Limitations

This study has limitations. Firstly, the TCR database does not include personal-level data for cancer patients, leading to a lack of information on factors such as tumor size, stages, or tissue subtypes. The utilization of individual data could allow us to monitor changes in the oncological status of individuals post-thyroid cancer screening, providing insights into cancer staging and mortality rates. We could investigate whether patients at different stages undergo ultrasound screening and if their mortality rates differ. Additionally, utilizing individual-level data, we can control for potential confounding factors during our analysis. This may also comprehensively evaluate the impact of overdiagnosis on the increasing thyroid cancer incidence rates in Taiwanese females. Second, to investigate the causal association between thyroid cancer and fertility, we employed the concept of causal temporality, utilizing data from cohorts with identical birth years. We conducted a correlation analysis between the birth rates of individuals aged 20–29 and the thyroid cancer incidence rates in the 50–54 age group, which yielded statistically significant results. Simultaneously, we selected countries from different regions and compared the birth rates from 1960, 1980, and 2010 with age-standardized incidence rates for the periods 1973–1977, 1993–1997, and 2013–2017, revealing a consistent correlation. While the analyses mentioned earlier were carried out at the group level, it is imperative for future investigations to incorporate a research design at the individual level. To enhance our understanding and substantiate the association between thyroid cancer and fertility, there is a need for additional individual-level data. Furthermore, integrating results from molecular or biological research will contribute significantly to a more comprehensive exploration of this relationship.

## 5. Conclusions

Our results show an increasing thyroid cancer incidence in females in Taiwan, with overdiagnosis being an important reason for the increase. Notably, we discovered a strong negative correlation between fertility and thyroid cancer rates. Many risk factors for thyroid cancer exist in Taiwan, but with the exception of overdiagnosis, childbirth, and plastic exposure, other risk factors have not risen much. However, thyroid cancer incidence in females continues to rise rapidly, especially among those aged 50–54. Therefore, we speculate that changes in fertility rates have impacted thyroid cancer incidence, a finding that is rarely discussed in previous studies. The mechanisms behind this finding deserve to be explored in future studies. Decline in fertility is a global issue, and thyroid cancer incidence is increasing at different rates in all countries. Therefore, this has important implications as a global demographic issue, and investigating changes in fertility is important for future thyroid cancer research. Future research will be necessary to estimate the impact of overdiagnosis on changes in thyroid cancer incidence, utilizing the relationship between incidence and mortality rates. Additionally, exploring the correlation between individual-level fertility and thyroid cancer could be undertaken.

## Figures and Tables

**Table 1:** Annual percent change (APC) in female thyroid cancer overall incidence over time by age, 30–89.

Characteristics	Joinpoint Segment Start Year	Joinpoint Segment End Year	APC (95% CI)		*p*-Value
Females	1995	2019	5.9	(5.3–6.5)	<0.001
By age					
30–34	1995	2019	4.4	(2.2–6.6)	0.008
35–49	1995	2019	5.8	(4.3–7.3)	0.001
40–44	1995	2019	6.2	(5.5–6.9)	<0.001
45–49	1995	2019	6.8	(5.5–8.1)	<0.001
50–54	1995	2019	6.8	(6.1–7.5)	<0.001
55–59	1995	2019	6.6	(4.8–8.5)	0.001
60–64	1995	2019	6.2	(5.3–7.1)	<0.001
65–69	1995	2019	5.1	(3.0–7.3)	0.005
70–74	1995	2019	4.0	(2.2–5.8)	0.005
75–79	1995	2019	2.9	(1.9–3.9)	0.002
80–84	1995	2019	0.7	(−0.9–2.3)	0.248
85–89	1995	2019	2.3	(−0.9–5.6)	0.111

## Data Availability

The datasets generated and/or analyzed during the current study are not publicly available in accordance with the policy of the Health and Welfare Data Science Center, Ministry of Health and Welfare, Taiwan, but are available from the corresponding author upon reasonable request.

## Supplementary Materials

The following supporting information can be downloaded at: https://www.mdpi.com/article/10.3390/life14070809/s1, Figure S1: (a) Correlation between age-specific thyroid cancer incidence rates and cohort fertility rates in Taiwanese females aged 20–24 (b) Correlation between age-specific thyroid cancer incidence rates and cohort fertility rates in Taiwanese females aged 25–29; Figure S2: Incidence rates of thyroid cancer and fertility rates in selected countries; Table S1: Age-specific fertility rates in Taiwanese females, 1950–1999.
