# Supplementary material for: Secular Increasing Trends in Female Thyroid Cancer Incidence in Taiwan
Source: Life (Basel). 2024 Jun 26;14(7):809. doi: 10.3390/life14070809 (PMC11278399; doi:10.3390/life14070809)
Supplement: Supplementary file 1 [file life-14-00809-s001.zip › life-3066019-supplementary.pdf]

Supplementary Materials

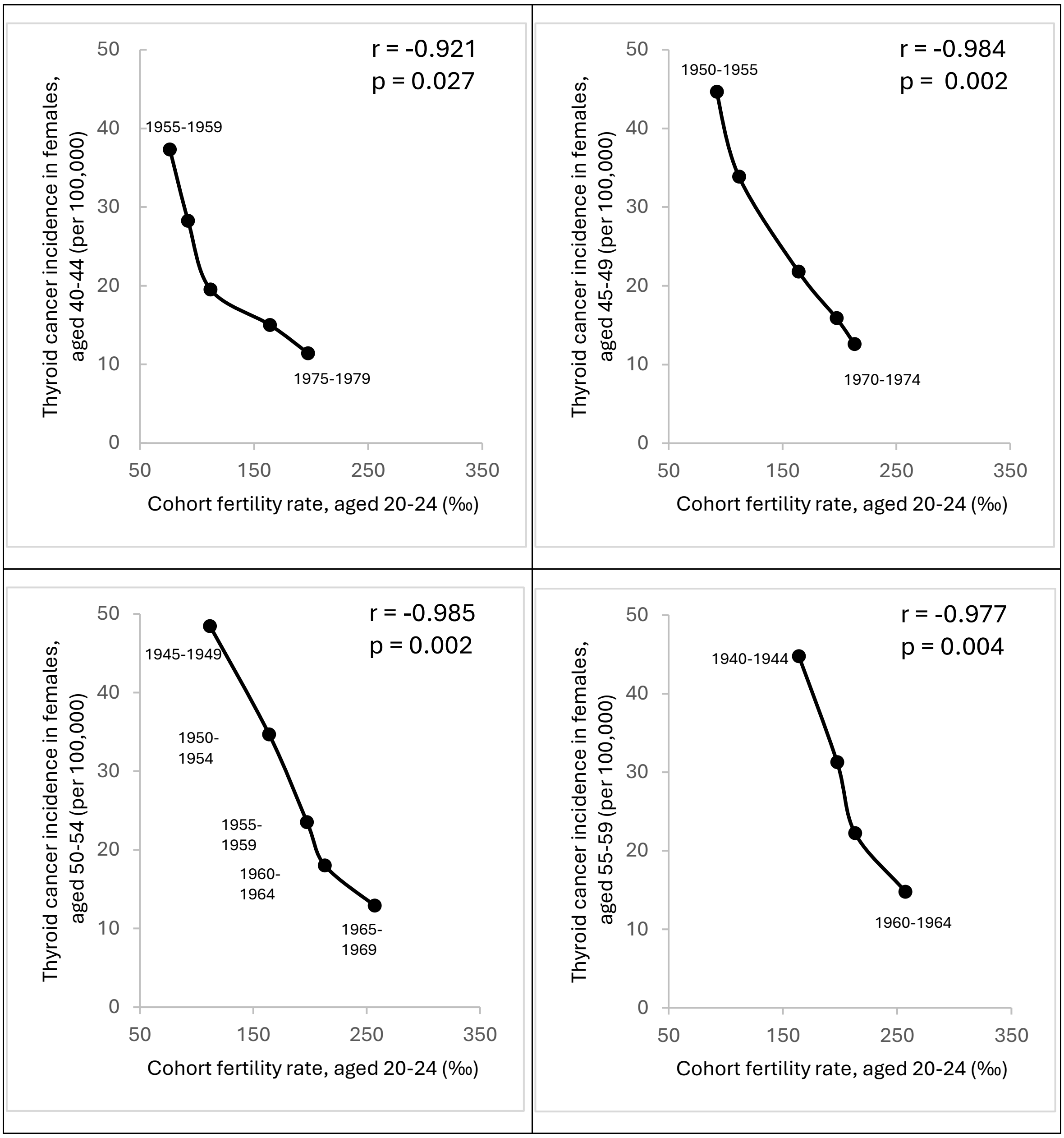

Supplementary Figure 1. (a) Correlation between age-specific thyroid cancer incidence rates and cohort fertility rates in Taiwanese females aged 20-24.

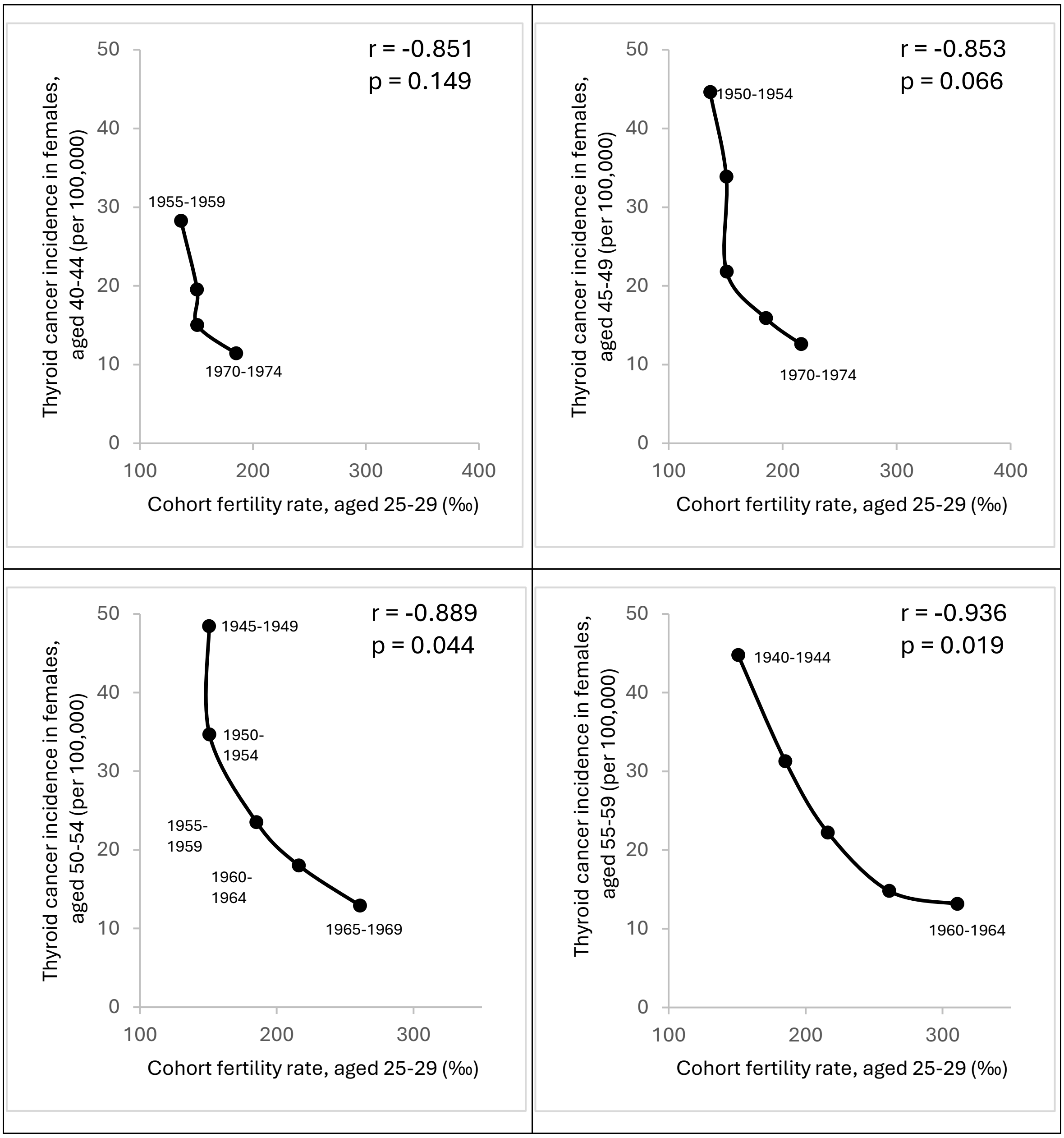

Supplementary Figure 1. (b) Correlation between age-specific thyroid cancer incidence rates and cohort fertility rates in Taiwanese females aged 25-29.

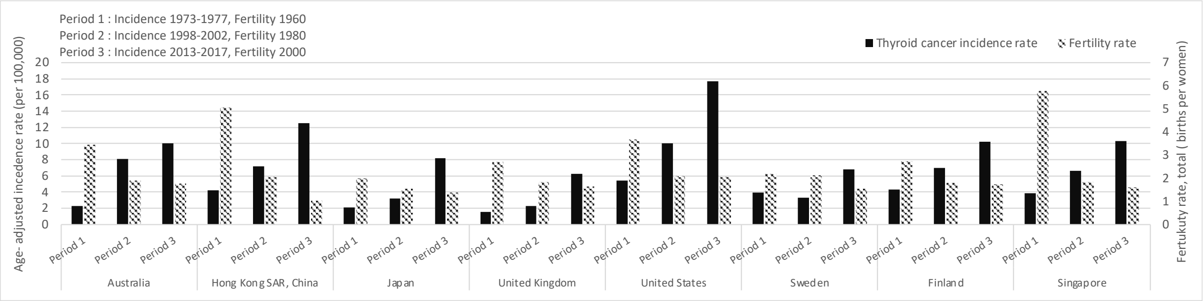

Supplementary Figure 2. Incidence Rates of Thyroid Cancer and Fertility Rates in Selected Countries

Supplementary Table 1. Age-specific fertility rates in Taiwanese females, 1950-1999

year	age-specific fertility rate（‰, per mil）					
	15-19	20-24	25-29	30-34	35-39	40-44
1950-1954	55.6	266.6	331.8	291.6	214.6	113.8
1955-1959	47	258.4	335.2	283.4	205.4	95.2
1960-1964	43.2	252.4	337	236	145.8	65.4
1965-1969	39.2	257.2	310.8	170.6	78.4	31.2
1970-1974	35.2	213.2	261	118.8	44.2	14.2
1975-1979	36	197.4	216.2	77.4	23	6.2
1980-1984	28.4	164	185.2	65.4	14.2	3
1985-1989	17	111.8	150.8	57.4	12.8	2
1990-1994	17	92.2	150.6	72.6	16.6	2
1995-1999	15.2	76.2	136.4	81.6	21	2.6
